# Novel *JAG1* variants leading to Alagille syndrome in two Chinese cases

**DOI:** 10.1038/s41598-024-52357-0

**Published:** 2024-01-20

**Authors:** Xiufang Feng, Jiangyuan Ping, Shan Gao, Dong Han, Wenxia Song, Xiaoze Li, Yilun Tao, Lihong Wang

**Affiliations:** 1 Department of Pediatrics, Changzhi Maternal and Child Health Care Hospital, Changzhi, Shanxi China; 2 Medical Genetic Center, Changzhi Maternal and Child Health Care Hospital, Changzhi, Shanxi China; 3 Obstetrics Department, Changzhi Maternal and Child Health Care Hospital, Changzhi, Shanxi China; 4 Precision Medicine Research Division, Changzhi Maternal and Child Health Care Hospital, Changzhi, Shanxi China

**Keywords:** Genetics, Diseases, Neurology

## Abstract

Alagille Syndrome (ALGS) is a complex genetic disorder characterized by cholestasis, congenital cardiac anomalies, and butterfly vertebrae. The variable phenotypic expression of ALGS can lead to challenges in accurately diagnosing affected infants, potentially resulting in misdiagnoses or underdiagnoses. This study highlights novel *JAG1* gene mutations in two cases of ALGS. The first case with a novel p.Pro325Leufs*87 variant was diagnosed at 2 months of age and exhibited a favorable prognosis and an unexpected manifestation of congenital hypothyroidism. Before the age of 2, the second patient was incorrectly diagnosed with liver structural abnormalities, necessitating extensive treatment. In addition, he exhibited delays in language acquisition that may have been a result of SNAP25 haploinsufficiency. The identification of ALGS remains challenging, highlighting the importance of early detection and genetic testing for effective patient management. The variant p.Pro325Leufs*87 is distinct from reported variants linked to congenital hypothyroidism in ALGS patients, thereby further confirming the clinical and genetic complexity of ALGS. This emphasizes the critical need for individualized and innovative approaches to diagnosis and medical interventions, uniquely intended to address the complexity of this syndrome.

## Introduction

Alagille syndrome (ALGS) is a rare and complex genetic disorder characterized by a wide spectrum of clinical manifestations affecting multiple organ systems, including the liver, heart, bones, eyes, face, and kidneys. The hallmark features of this condition are a reduced number of bile ducts and the occurrence of cholestasis. Congenital cardiac defects, such as pulmonary artery stenosis and tetralogy of Fallot, are commonly encountered in individuals. Additionally, these patients may present with butterfly vertebrae, posterior embryotoxon, renal abnormalities and identifiable facial features^1–3^.

The initial characterization of this disorder can be traced back to 1969 when Daniel Alagille first described its clinical manifestations^4^. It wasn't until 1997 that the underlying cause of ALGS was identified as a heterozygous mutation in the *JAG1* gene (MIM 601920), which is located on chromosome 20p12.2 and encoding the Notch receptor^1,5^. Subsequent investigations have revealed that ALGS is associated with dysfunction in the Notch signaling pathway, and pathogenic variations in the NOTCH2 gene (MIM 610275), another Notch signaling receptor, have also been implicated^3,6^.

Given its rarity, with estimated occurrences ranging from approximately 1 in 30,000 to 1 in 50,000 live births^7^, ALGS poses diagnostic challenges due to the diverse clinical manifestations and variability in its presentation. Consequently, misdiagnosis or insufficient recognition can lead to inappropriate therapeutic interventions. To address these issues, our study conducted a comprehensive comparative analysis, building on previous research, to gain a deeper understanding of the genetic context and clinical features of this rare disorder. Furthermore, our study aimed to broaden the understanding of the mutation spectrum associated with ALGS by conducting an investigation into the clinical and genomic features of two patients exhibiting a connection to the *JAG1* gene.

## Materials and methods

### Ethical statement

The subjects in this study are two male individuals who presented with cholestasis during the neonatal period. Blood samples were obtained from both patients and their parents to conduct further genetic testing. This study was approved by the Clinical Research Ethics Committee of Changzhi Maternal and Child Health Care Hospital. Written informed consent was obtained from the parents/legal guardian of the patients included in this study.

### Genetic analysis

Whole-exome sequencing (WES) performed for each patient using a whole exome capture kit (MyGenostics Inc., Beijing, China) according to the manufacturer’s protocol^8,9^. Sequencing of captured DNA fragments was carried out with 150 bp paired-end reads on Illumina HiSeq × Ten platforms. After sequencing, clean reads were mapped to the UCSC hg19 human reference genome using the parameter BWA of Sentieon Software (https://www.sentieon.com/). The SNP and InDel variants were identified and filtered through the utilization of the Genome Analysis Toolkit. The information would subsequently be converted to VCF format. The variants underwent additional annotation using the ANNOVAR software (http://annovar.openbioinformatics.org/en/latest/). They were linked to various databases, including gnomAD (730,947 exomes and 76,215 genomes), Inhouse (MyGenostics, 160,174 exomes), and HGMD. Additionally, SIFT, PolyPhen-2, MutationTaster, and GERP +  + were utilized to predict the variants. The evaluation of mutation pathogenicity was conducted in adherence to the guidelines set forth by the American College of Genetics and Genomics (ACMG)^10^. Sanger sequencing was utilized to validate the candidate variable sites. Copy number variants (CNVs) were elevated by measuring the relative NGS read depth at target positions by CNVkit (https://cnvkit.readthedocs.io/en/stable/) using the algorithm described previously^11,12^.

### Literature review

The evaluation involved relevant publications retrieved using search terms "Alagille syndrome" or "JAG1", which provided insights into the specific molecular genetic characteristics of ALGS. The collated clinical and genetic information, along with patient outcomes, were compiled from these articles and are summarized in Supplementary Tables 1 and 2.

### Ethics approval and consent to participate

Informed consent was obtained from the patients for drafting the manuscript and the research follows ethical guidelines.

## Results

### Clinical features of Case 1

The proband was delivered by cesarean section at 38 weeks gestation with intrauterine growth restriction. His birth weight was 2250 g (< 1st percentile), his body length measured 45 cm (3rd percentile), and his head circumference was 33 cm (50th percentile). Due to his low birth weight, he was admitted to the neonatology department for one week after birth. At the age of 26 days, he was readmitted for therapy due to jaundice and hypothyroidism. Total bilirubin levels were found to be elevated (189.5umol/L). Thyroid function tests revealed abnormal results, with TSH of 24.638mIU/L, TT4 of 57.49 nmol/L, TT3 of 1.22 nmol/L, FT4 of 9.05 pmol/L, and FT3 of 4.77 pmol/L (Table 1). Additional tests, including an ultrasound of the thyroid and an X-ray of the bone age, revealed no abnormalities. During fasting, abdominal ultrasonography revealed hepatomegaly, but no kidney abnormalities.

**Table 1:** Results of laboratory investigations in Case 1.

Age (months)	1	3	4	5	6	9	10
ALT (U/L) (8–71)	–	356.9	842.8	711.5	599.7	602.0	588.9
AST (U/L) (21–80)	–	248.2	544.7	470.2	473.3	445.2	462.2
GGT (U/L) (6–31)	–	498.7	208.8	277.7	102.1	43.0	241.9
ALP (U/L) (106–420)	–	938.0	1195.9	852.8	470.5	412.5	444.1
TSH (IU/L) (0.75–5.75)	24.638	4.133	–	5.958	7.232	9.160	5.937
TT4 (nmol/L) (69.71–163.95)	57.49	–	–	88.93	92.44	114.35	151.23
TT3 (nmol/L) (0.92–2.38)	1.22	–	–	1.22	–	1.75	1.62
FT4 (pmol/L) (9.40–19.54)	9.05	10.68	–	10.71	–	12.63	12.42
FT3 (pmol/L) (4.21–8.16)	4.77	5.88	–	4.53	–	6.11	5.75

There were no abnormalities found in the liver, pancreas, or spleen on ultrasound examination. At two months of age, a histological examination of the liver "after liver biopsy" revealed grade 1 inflammation and stage 1 fibrosis, as well as an absence of intrahepatic bile ducts. The elasticity of the liver was measured at 6.56 Kpa, and the matrix metalloproteinase-7 (MMP-7) level was 8.

A chest X-ray taken at the age of 5 months shows butterfly vertebrae at the T3, T6, and T8 levels. A cardiac ultrasound also revealed the presence of a patent foramen ovale. A thorough ophthalmologic examination revealed no anomalies. The patient exhibited considerable failure to thrive, as evidenced by his weight of 6000 g (1st percentile), body length measuring 55 cm (1st percentile), and head circumference of 40 cm (1st percentile). By 7 months of age, his weight had risen to 7000 g (3rd percentile), his body length had reached 65 cm (5th percentile), and his head circumference had remained at 42 cm (1st percentile).

When the patient was 10 months old, the chronic jaundice was still present. Liver enzyme readings remained high. The facial features of the patient resembled those with ALGS in that he had a prominent forehead with a pointed chin giving the face an inverted triangle appearance. He had hypertelorism, deep-set eyes, and a depressed nasal bridge.

The father of the patient has a history of jaundice and aortic arch stenosis. Grandfather and grandmother of the patient are both healthy. The other family members do not exhibit any symptoms or indications of ALGS.

### Clinical features of Case 2

The patient, a male, is the first-born child of healthy, non-consanguineous Chinese parents who experienced two spontaneous abortions before his birth. He was born prematurely at 34 weeks, weighing 1690 g (5th percentile), and measuring 42 cm (25th percentile) in length. He was also admitted to the neonatology department with hyperbilirubinemia (3 times the upper limit of normal) secondary to neonatal cholestasis, feeding intolerance, and infectious enteritis. The laboratory findings are presented in Table 2.

**Table 2:** Results of laboratory investigations in Case 2.

Age (months)	1	2	6	7	9	19	20	21	23	24	25	33
ALT (U/L) (8–71)	100.0	84.8	175.4	203.9	192.0	161.9	106.1	192.6	276.7	550.9	159.9	222.8
AST (U/L) (21–80)	224.8	144.2	125.1	200.5	174.7	163.6	187.5	174.7	168.0	372.0	200.1	162.6
GGT (U/L) (6–31)	1043.6	1305	708.4	884.4	548.8	677.0	719.8	548.8	761.3	539.2	1108.9	383.5
ALP (U/L) (106–420)	320.0	515.3	1258.2	776.2	616.9	803.6	781.9	616.9	728.0	939.9	847.1	712.3

**Figure 1:**
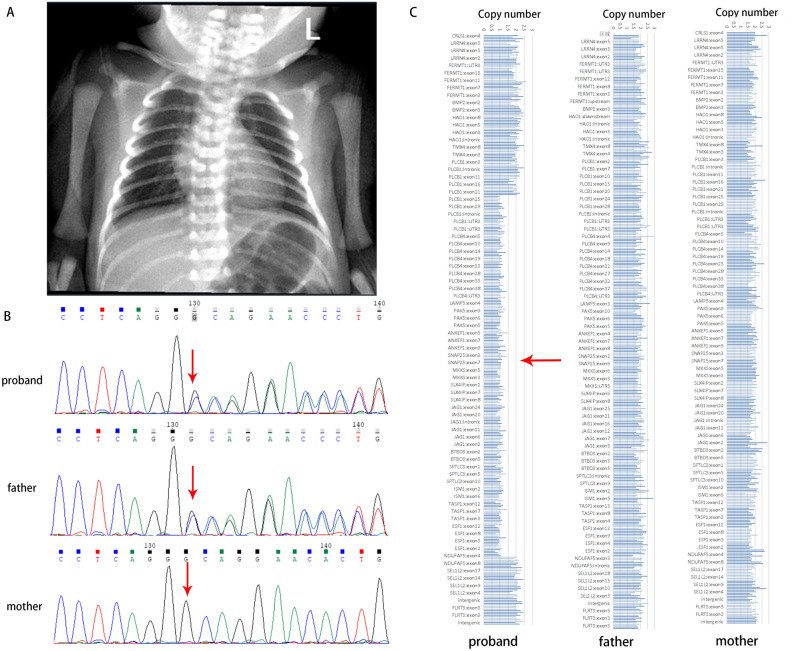
Clinical and genetic feature of the two patients. (**A**) Chest X-ray of Case 2 reveals butterfly vertebra. (**B**) Sequencing results for Case 1 and their family members. Sanger sequencing revealed that the variant c.974delC (p.Pro325Leufs*87) (red arrow) was paternally inherited. (**C**) Copy number variation (CNV) analysis derived from WES data for Case 2, illustrating a 5.04 Mb deletion (chr20:8731424-13773919, red arrow) spanning the region 20p12.3p12. The Morbid genes are highlighted in red font.

At one month of age, the patient underwent an ultrasound examination that revealed an underdeveloped right kidney measuring 1.8 × 0.8 cm (< 1st percentile). The liver and spleen appeared normal in the imaging findings. Additionally, a heart ultrasound indicated the presence of a persistent patent foramen ovale, and a chest X-ray showed butterfly vertebrae (Fig. 1A). Despite receiving biliary flushing treatment at three months of age, the patient's liver enzyme levels continued to remain elevated beyond the normal range.

At 4 months, the patient was able to elevate their head; at 7 months, they were able to sit independently; and by 11 months, they were able to stand independently. At 1.5 years of age, the patient was able to walk independently. The right kidney measured approximately 2.6 × 0.8 cm (< 1st percentile), indicating renal dysplasia. The patient exhibited facial features consisting of an elongated long nose, large ears, deep set eyes, and there was an inverted triangle appearance of the face. At 2 years and 3 months of age, the patient experienced seizures without any evidence of fever, exhibiting symptoms such as mouth puckering, eye blinking, and limb quivering. Electroencephalogram (EEG) results at baseline were normal. No abnormalities were detected during the ophthalmologic examination.

The individual exhibited a delay in the acquisition and progression of speech and language skills. At the age of two, he began using two-word sentences. By age three years his speech was delayed and limited to two- or three-word phrases. In addition, he exhibited failure to thrive, with a height of 88 cm (< 1st percentile) and a weight of 11 kg (< 1st percentile) at the age of three years.

### Genetic analysis

Case 1 underwent WES testing at the age of 2 months. The results revealed a paternally inherited variant in *JAG1* (NM_000214), specifically c.974delC (p.Pro325Leufs*87) (Fig. 1B). This genetic variant was absent from the general population and had not been previously described in the scientific literature. It was predicted using the NMD Prediction Tool (https://nmdpredictions.shinyapps.io/shiny) that it would result in an early termination codon and nonsense-mediated degradation of the mRNA. The p.Pro325Leufs*87 variation was classified as "likely pathogenic" according to the ACMG guideline (PVS1 + PM2_Supporting).

WES testing was performed when Case 2 was 2 years old, which identified a de novo heterozygous deletion of 5.04 Mb in the 20p12.3p12.1 region (chr20:8731424-13773919) (Fig. 1C). This deletion encompassed approximately 15 known protein-coding genes, of which *JAG1*, *SNAP25*, *MKKS*, *NDUFAF5*, *PLCB1*, *PLCB4*, and *TASP1* genes have been previously linked to various human disorders (Supplementary Table 3).

The ALGS diagnosis of the two patients was confirmed by analyzing the molecular genetics data and clinical findings.

## Discussion

Evaluation of neonatal cholestasis is undeniably a complex and crucial procedure, as it is associated with over a hundred different conditions, making it difficult to accurately diagnose and manage^13^. Currently, therapies for neonatal cholestasis concentrate primarily on managing symptoms and addressing complications, as opposed to focusing on the underlying cause^14,15^. In some instances, ALGS has been misdiagnosed as biliary atresia, resulting in inappropriate surgical management such as a Kasai procedure. The Kasai procedure is contraindicated in ALGS as it may result in higher rates of mortality and liver transplantation in patients with ALGS^16,17^. Therefore, expeditious and accurate genetic prompt diagnosis of neonatal cholestasis is crucial. Nonetheless, among 889 individuals diagnosed with ALGS through molecular testing (Supplementary Table 1), the average age at diagnosis was 7.28 ± 12.61 years, indicating a significant delay compared to the onset age (2.4387 ± 6.8176 years) (p < 0.001). This notable delay underscores a common problem of delayed diagnosis for ALGS, particularly in China, where symptoms may manifest long before the condition is recognized and confirmed^16,18^. In this study, Case 2 received a confirmed diagnosis at the age of two years. However, the delayed diagnosis of the disease consumed a substantial amount of the patient's time and resources, as he had to visit various hospitals in different cities across China to seek solutions for the condition's manifestations. In addition, he underwent procedures such as biliary drainage, which may not have adequately addressed the root cause. Conversely, Case 1 received systematic treatment after a firm diagnosis at 2 months of age, leading to a more positive outlook in terms of acceptance, compliance, and prognosis. Indeed, the rarity of ALGS and limited awareness among many Chinese physicians pose challenges in accurately diagnosing the condition based solely on clinical symptoms. Even when neonatal patients present with cholestasis, congenital cardiac abnormalities, or skeletal deformities, healthcare providers face significant obstacles in establishing a link with ALGS and implementing the appropriate treatment and management strategies. Nonetheless, the introduction of next-generation sequencing (NGS) has substantially aided in the quick diagnosis of disorders during the neonatal period^19,20^.

To date, 604 distinct pathogenic (or likely pathogenic) variants in the *JAG1* gene have been reported in ALGS patients (Supplementary Table 2). These variants included 233 frameshift mutations, 120 nonsense mutations, 118 missense mutations, 80 splicing site mutations, 50 large deletions, and 3 irregular variants. The distribution of these variations in the *JAG1* gene was not uniform. Most variants were found in exons (577 out of 604, 95.53%), with a concentration of variants found in exons 2, 4, 6, 16, 23, and 24with a substantial concentration seen in exonic areas (577 out of 604, 95.53%), especially exons 2, 4, 6, 16, 23, and 24.

A review of AGLS cases reported in the literature highlighted that, 93.8% (531 out of 566) exhibited liver abnormalities, with cholestasis being the most prevalent sign. Heart abnormalities, such as atrial septal defect, pulmonary stenosis, Tetralogy of Fallot, and ventricular septal defect, were observed in 90.2% (542 out of 601) of the patients. Distinctive facial features were present in 456 out of 519 individuals (87.9%). Furthermore, 57 out of 87 (65.5%) research participants exhibited spinal anomalies, and 95 out of 191 subjects (49.7%) had posterior embryotoxon. Only 87 out of 301 patients (28.9%) had renal issues.

**Figure 2:**
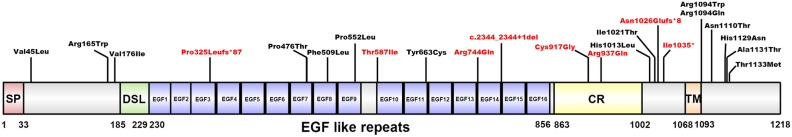
Reported variants in the *JAG1* gene in the patients with CH. The domain of the *JAG1* gene is shown including signal peptide (SP, residues 1–33), DSL (residues 185–229), EGF like repeats (residues 230–856), cysteine‐rich region (CR, residues 863–1002) and trans-membrane motif (TM, residues 1068–1093). Variants observed in patients manifesting both ALGS and CH symptoms are highlighted in red.

Research findings suggest that the Notch signaling system may be involved in the development of the thyroid gland^17^. However, congenital hypothyroidism (CH) remains uncommon in individuals with *JAG1* mutations, documented in a limited 27 cases, of which 18 solely exhibited CH symptoms (Supplementary Table 4)^21–24^. Among patients manifesting both ALGS and CH symptoms, six variants (p.Thr587Ile, p.Arg744Gln, c.2344_2344 + 1del, p.Cys917Gly, p.Asn1026Glufs8, and p.Ile1035) were identified (Fig. 2), after excluding mutations in known CH candidate genes. Conversely, among those solely displaying CH symptoms, 16 missense mutations were detected. Despite three of these variants not present in the gnomAD database, most exhibited a minor allele frequency above 0.00001, contrasting the typical inheritance pattern linked to ALGS. Surprisingly, among these 16 missense mutations, 14 were categorized as likely benign or benign, and 2 were uncertain significance. These findings imply: (i) *JAG1* missense mutations could be linked to CH, although with limited penetrance, resembling the reduced penetrance seen in some autosomal dominant genetic diseases^25,26^; (ii) Loss-of-function (LOF) variations in *JAG1* might lead to a hypothyroid condition, as evidenced in zebrafish studies^21^; (iii) *JAG1* might function as a gene modifier, signifying minor functional impairments resulting from the coexistence of rare alleles in particular thyroid genes^23,24^; and (iv) unidentified factors might have contributed to CH, resulting in the simultaneous occurrence of cardiovascular abnormalities akin to those seen in ALGS patients^27^.

Out of the 604 *JAG1* variants identified, the majority—precisely 483 out of 604 (79.97%)—were LOF variants. Among these, only three LOF variants were specifically noticed in patients exhibiting symptoms of both ALGS and CH. These variants potentially impact the trans-membrane motif (residues 1068–1093) or the cysteine-rich region (residues 863–1002): c.2344_2344 + 1del (perturbing exon 18 and intron 18), c.3075dup (p.Asn1026GlufsTer8, located in exon 25), and c.3103del (p.Ile1035Ter, situated in exon 25). In a unique divergence from previous observations, Case 1 carried a LOF mutation in exon 7, specifically p.Pro325Leufs*87 that also affected the EFG like repeats (230–856 residues), also presents a significant challenge in unraveling the pathogenesis of CH in ALGS patients. The co-occurrence of this CH revelation and typical ALGS symptoms highlights the fortuitous nature of CH detection. The fluctuating TSH levels that occurred during treatment as a result of difficulties in medication dosage control caused by growth and developmental delays highlight the critical need for thyroid function surveillance in ALGS patients with CH. It is critical to perform regular growth and developmental evaluations in order to promptly modify drug concentrations and improve treatment results. Given the uncertainty surrounding how JAG1 affects CH development, it might be beneficial to include routine thyroid function assessments for ALGS patients, particularly those experiencing failure to thrive.

Language developmental impairments are infrequently observed in patients with ALGS, although there was a perceptible delay in Case 2. Specifically, by the age of three, expressive language development in this case was limited to two- or three-word phrases. Although there is a known association between mutations in the *JAG1* gene and craniofacial deformities, the precise implications of these abnormalities on oral-motor function and speech capabilities have yet to be comprehensively elucidated. Several explanations are plausible for the observed language impairments: Firstly, chronic liver disease might contribute to delayed language skill development^28^. Secondly, the significant deletion might be associated with developmental delays^29^. Thirdly, the *SNAP25* gene within the deletion area stands as a prime candidate. This is supported by the presence of the nonsense variants c.520C > T (p.Gln174*) and c.589C > T (p.Gln197*) in SNAP25, associated with epilepsy and speech difficulties^30,31^. The correlation is additionally reinforced by the existence of nonsense mutations, c.164_252del (p.Glu55Valfs9) and c.464del (p.Gly155fs84), which the Clinvar database has classified as potentially or actually pathogenic. Further investigation has demonstrated a correlation between SNAP25 dysfunction and postponed psychomotor development using a *SNAP25* knockout mouse model. Despite these connections, there is insufficient evidence to substantiate the hypothesis that these mutations endure nucleotide modification deletion (NMD). After ruling out alternative genes within the deleted region, *SNAP25* continues to be the most prominent candidate. Additionally, the occurrence of seizures in Case 2 hints at a potential connection with *SNAP25*. However, additional investigation is required in order to comprehensively comprehend these possible correlations.

## Conclusions

In conclusion, this study has revealed novel heterozygous mutations within the *JAG1* gene. The initial patient displayed characteristic signs of ALGS in addition to CH. Nevertheless, the distinct mutation p.Pro325Leufs*87 found in the *JAG1* gene sets it apart from previously documented instances of CH. This discovery implies the potential existence of additional locations or types of genetic abnormalities in the *JAG1* gene that may be linked to CH, emphasizing the importance of diligent monitoring of thyroid function in individuals diagnosed with ALGS. The second case involved the identification of language developmental impairments, which could potentially be linked to the haploinsufficiency of the *SNAP25* gene located within the deletion region. Furthermore, we thoroughly reviewed the literature and documented *JAG1* variants, thereby contributing to the progression of understanding pertaining to ALGS. This study highlights the significance of genetic testing and comprehensive clinical assessments in improving the time to diagnosis and the clinical management and treatment of this complex syndrome.

### Supplementary Information

Supplementary Table 1.

Supplementary Table 2.

Supplementary Table 3.

Supplementary Table 4.

## Data Availability

The datasets used and/or analysed during the current study available from the corresponding author on reasonable request.

## Abbreviations

ALGS: Alagille syndrome
APTT: Extended activated partial thromboplastin time
CH: Congenital hypothyroidism
CNVs: Copy number variants
NGS: Next-generation sequencing
PT: Prolonged prothrombin time
WES: Whole exome sequencing
